# Effects of Mean Normal Stress and Microstructural Properties on Deformation Properties of Ultrahigh-Strength TRIP-Aided Steels with Bainitic Ferrite and/or Martensite Matrix Structure

**DOI:** 10.3390/ma17143554

**Published:** 2024-07-18

**Authors:** Koh-ichi Sugimoto, Shoya Shioiri, Junya Kobayashi

**Affiliations:** 1 Graduate School of Science and Technology, Shinshu University, Nagano 380-8553, Japan; mellow.pretty8330@gmail.com; 2 Graduate School of Science and Engineering, Ibaraki University, Hitachi 316-8511, Japan; junya.kobayashi.jkoba@vc.ibaraki.ac.jp

**Keywords:** advanced ultrahigh-strength steel, TRIP-aided steel, deformation property, mean normal stress, microstructural property

## Abstract

The effects of mean normal stress on the deformation properties such as the strain-hardening, strain-induced martensite transformation, and micro-void initiation behaviors of low-carbon ultrahigh-strength TRIP-aided bainitic ferrite (TBF), bainitic ferrite/martensite (TBM), and martensite (TM) steels were investigated to evaluate the various cold formabilities. In addition, the deformation properties were related to the microstructural properties such as the matrix structure, retained austenite characteristics, and second-phase properties. Positive mean normal stress considerably promoted strain-induced martensite transformation and micro-void initiation, with an increased strain-hardening rate in an early strain range in all steels. In TM steel, the primary martensite matrix structure suppressed the micro-void initiation through high uniformity of a primary martensite matrix structure and a low strength ratio, although the strain-induced transformation was promoted, and a large amount of martensite/austenite constituent or phase was contained. A mixed matrix structure of bainitic ferrite/primary martensite in TBM steel also suppressed the micro-void initiation because of the refined microstructure and relatively stable retained austenite. Promoted micro-void initiation of TBF steel was mainly promoted by a high strength ratio.

## 1. Introduction

The third-generation advanced ultrahigh- and high-strength steels (AHSSs) have been developed for lightening automobiles and improving crash safety [1,2,3]. The third-generation AHSSs are classified into the following three groups.

“Group L”: TRIP-aided bainitic ferrite (TBF) steel [4,5], carbide-free bainitic (CFB) steel [6,7], and duplex-type medium Mn (D-MMn) steel [8,9].

“Group M”: TRIP-aided bainitic ferrite/martensite (TBM) steel [10] and quenching and partitioning (Q&P) steel [11,12]

“Group H”: TRIP-aided martensite (TM) steel [10] and martensite-type medium Mn (M-MMn) steel [13].

**Figure 1:**
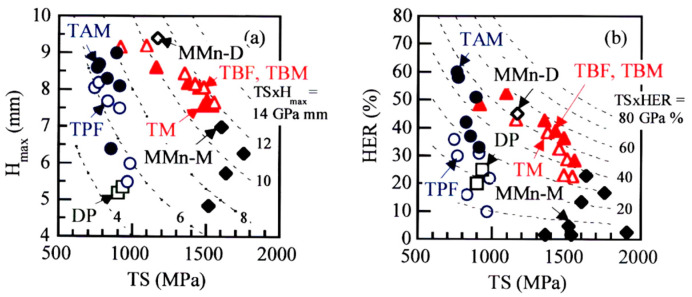
(**a**) Maximum stretch height (*H*_max_), tensile strength (TS), and (**b**) hole expansion ratio (*HER*); TS relations at room temperature in low-carbon 0.15C-0.25Si-1.70Mn ferrite-martensite dual-phase (DP) steel [14], 0.2C-(1.0-2.5)Si-(1.0-2.0)Mn TRIP-aided polygonal ferrite (TPF) and TRIP-aided annealed martensite (TAM) steels [14], 0.20C-1.5Si-1.5Mn-0.05Nb TRIP-aided bainitic ferrite (TBF), bainitic ferrite/martensite (TBM), and martensite (TM) steels [10], and 0.21C-1.50Si-4.94Mn duplex type (D-MMn) and martensite-type medium Mn (M-MMn) steels [8,13]. This figure is redrawn by using the results of Refs. [8,10,13,14]. The DP, TPF, and TAM steels belong to the first-generation AHSSs.

Any group of AHSSs contains a certain amount of metastable-retained austenite. The AHSSs of “Group L” have a tensile strength of less than 1.0 GPa and a matrix structure of bainitic ferrite. Only D-MMn steel has an annealed martensite matrix structure, in the same way as the first-generation AHSS such as TRIP-aided annealed martensite (TAM) steel [14]. On the other hand, the AHSSs of “Group H” have a tensile strength of higher than 1.5 GPa and a matrix structure of primary martensite. The AHSSs of “Group M” have a tensile strength between “Group L” and “Group H” steels and a mixed matrix microstructure of bainitic ferrite and primary martensite. The TBF, TBM, and TM steels possess extremely high cold stretch formability (maximum stretch height: *H*_max_) and stretch-flangeability (hole expansion ratio: HER), as shown in Figure 1 [8,10,13,14], in the same way as CFB [6,7] and Q&P steels [11,12]. The excellent formabilities are mainly associated with deformation properties such as high strain hardening, suppressed strain-induced martensite transformation, and suppressed micro-void initiation behaviors. As the formalities are measured under a different stress state or mean normal stress, it is essential to understand the effect of the mean normal stress on the deformation properties in the TBF, TBM, and TM steels, as well as the effect of microstructural properties. 

This research investigates the effect of the mean normal stress on the deformation properties at room temperature to evaluate the different cold formabilities in the low-carbon TBF, TBM, and TM steels. In addition, the deformation properties were related to the microstructural properties such as matrix structure, retained austenite characteristics, and martensite/austenite constitute (MA phase) properties.

## 2. Material and Methods 

**Figure 2:**
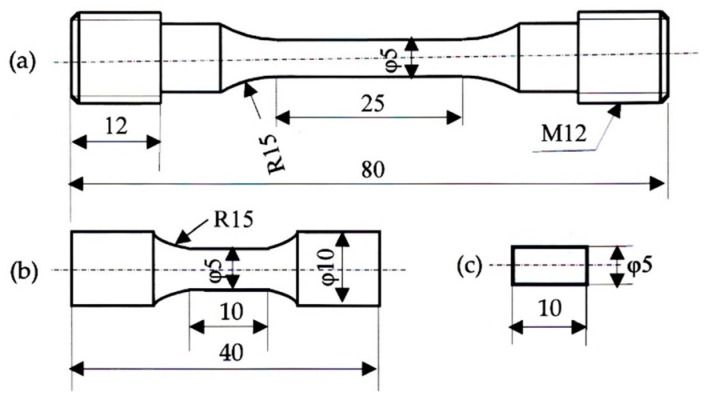
Dimensions (unit: mm) of (**a**) tensile, (**b**) torsional, and (**c**) compressive specimens.

**Figure 3:**
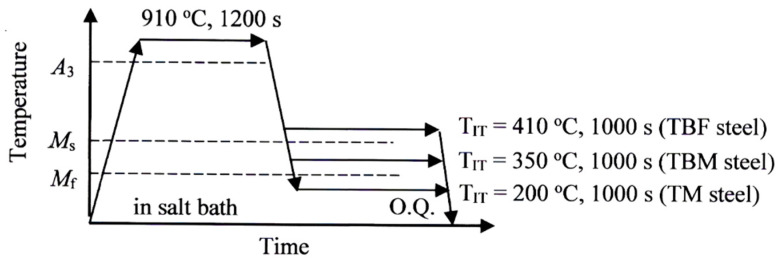
Heat treatment diagrams to produce TBF, TBM, and TM steels. The heat treatment was carried out in salt and oil baths. *T*_IT_: isothermal transformation temperature; O.Q: quenching in oil at 50 °C.

A steel slab of 100 kg with the chemical composition listed in Table 1 was manufactured using laboratory-based vacuum melting and then air cooling. Then, the slab was hot rolled to a 13 mm diameter at a finish temperature of 850 °C. Tensile specimens, torsional specimens, and compressive specimens, shown in Figure 2, were machined from the hot-rolled bars. To produce TBF, TBM, and TM steels with bainitic ferrite, bainitic ferrite/primary martensite mixture, and primary martensite matrix structures, respectively, three kinds of isothermal transformation (IT) treatments were carried out after austenitizing (Figure 3). These IT temperatures (*T*_IT_) are higher than *M*_s_, between *M*_s_ and *M*_f_, and lower than *M*_f_, respectively, at which the maximum retained austenite fractions are achieved.

The microstructure of the steels was observed at a 1/2 radius and a 1/2 height of the specimens before and after deformation using field-emission scanning electron microscopy (FE-SEM; JSM-6500F, JEOL Ltd., Akishima, Tokyo, Japan), which was performed using an instrument equipped with an electron backscatter diffraction system (EBSD; OIM system, TexSEM Laboratories, Inc., Prova, UT, USA). The beam area, beam diameter, beam step size, and acceleration voltage of the EBSD analysis were 40 × 40 μm^2^, 1.0 μm, 0.15 μm, and 25 kV, respectively. The specimens for the FE-SEM–EBSD analysis were first ground with emery paper (#1200), alumina powder, and colloidal silica. Finally, ion thinning was carried out.

The retained austenite characteristics of the steels were quantified using an X-ray diffractometer (RINT2000, Rigaku Co., Akishima, Tokyo, Japan). The surfaces of the specimens were electropolished after being ground with emery paper (#1200). The volume fraction of the retained austenite phase (*f*_γ_, vol.%) was calculated from the integrated intensity of the (200)α, (211)α, (200)γ, (220)γ, and (311)γ peaks obtained with X-ray diffractometry using Mo-Kα radiation [15]. The carbon concentration in the retained austenite (*C*_γ_, mass%) was estimated from the lattice constant of the (200)γ, (220)γ, and (311)γ peaks of the Cu-Kα radiation and the empirical equation proposed by Dyson and Holmes [16]. The X-ray half-width (HW) of (211)α peak of the Cu-Kα radiation was measured to relate to the equivalent plastic strain ($\bar{\varepsilon }$_p_) [17]. The above X-ray characteristics were measured using three or more samples.

The Vickers hardness (HV0.1) was measured using a Vickers microhardness tester (Shimadzu Co., DUH-201H, Kyoto, Japan) with a load of 0.98 N. Micro-void initiation behavior was observed by FE-SEM.

To develop various mean normal stress states, uniaxial tensile and compressive tests were conducted on a universal testing instrument (AD-10TD, Shimadzu Co., Kyoto, Japan) at 25 °C and a crosshead speed of 10 mm/min. Torsional tests were carried out on a torsion testing machine (AG-300kNXplus, Shimadzu Co., Kyoto, Japan) at 25 °C and a torsion rate of 10 deg./min. Three or more specimens were prepared to measure the mechanical properties.

Mean normal stress was defined by

*σ*_m_ = (*σ*_1_ + *σ*_2_ + *σ*_3_)/3 (1)

where σ_1_, σ_2_, and σ_3_ are principal stresses, respectively. An equivalent stress $\bar{\sigma }$ and equivalent strain $\bar{\varepsilon }$ were calculated using von Mises criterion [18], as follows,

(2) $$\bar{\sigma }=1/\surd 2\cdot [(\sigma_{x}-\sigma_{y}{)}^{2}+(\sigma_{y}-\sigma_{z}{)}^{2}+(\sigma_{z}-\sigma_{x}{)}^{2}+6({\tau_{xy}}^{2}+{\tau_{yz}}^{2}+{\tau_{xz}}^{2}){]}^{1/2}$$

(3) $$\bar{\varepsilon }=\surd 2/3\times [(\varepsilon_{x}-\varepsilon_{y}{)}^{2}+(\varepsilon_{y}-\varepsilon_{z}{)}^{2}+(\varepsilon_{z}-\varepsilon_{x}{)}^{2}+3/2\times ({\gamma_{xy}}^{2}+{\gamma_{yz}}^{2}+{\gamma_{zx}}^{2}){]}^{1/2}$$

where *σ*_i_, *ε*_i_ and *τ*_ij_, and *γ*_ij_ (i, j = x, y, z) represent normal stress, shear stress, normal strain, and shear strain in the X–Y–Z coordinate system, respectively. For tension tests, $\bar{\sigma }$ and $\bar{\varepsilon }$ of the necking region were calculated by

(4) $$\bar{\sigma }=\frac{P}{\pi d^{2}(1+\frac{2R}{d})ln(1+\frac{d}{2R})}$$

(5) $$\bar{\varepsilon }=2\ln\frac{d_{0}}{d}$$

where *P*, *d*, *d*_0_, and *R* are an applied load, a minimum diameter of the neck cross-section, an initial diameter of the specimen, and a radius of curvature of the neck profile, respectively [19].

## 3. Results

### 3.1. Microstructural Properties

**Figure 4:**
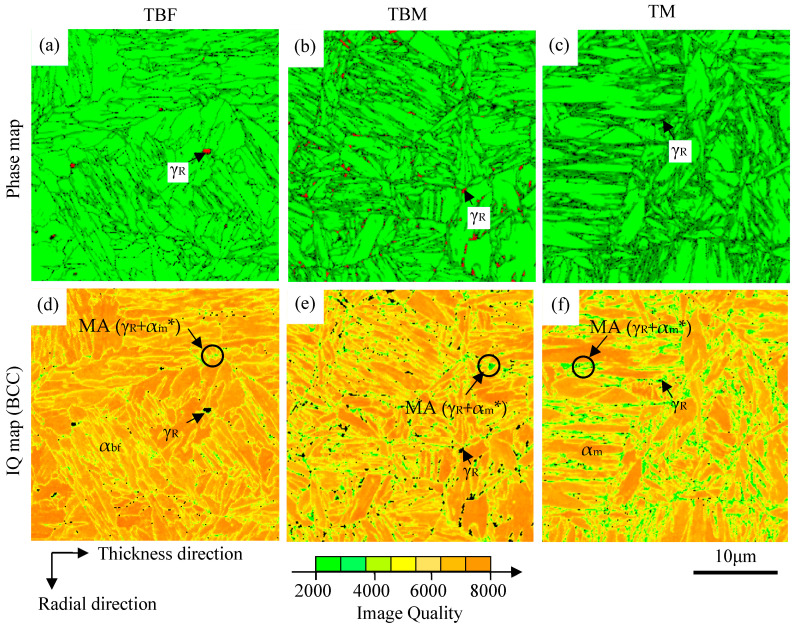
(**a**–**c**) Phase maps of BCC and FCC and (**d**–**f**) image quality (IQ) maps of BCC in TBF, TBM, and TM steels. α_bf_, α_m_, α_m_*, γ_R_, and MA are bainitic ferrite, primary martensite, secondary martensite, retained austenite, and martensite/austenite (MA) phase, respectively.

Figure 4 shows the microstructures of TBF, TBM, and TM steels observed in terms of FE-SEM-EBSD. In the same way as Ref. [10], the matrix microstructures of TBF, TBM, and TM steels are bainitic ferrite, bainitic ferrite/primary martensite, and primary martensite, respectively. As the second phase, carbon-enriched soft-retained austenite and a hard MA phase are contained in these steels. The martensite in the MA phase is carbon-enriched secondary martensite. TBM steel’s primary martensite fraction (*f*α_m_) can be estimated to be about 30 vol% using the following equation [20]:

*f*α_m_ = 1 − exp[−1.1 × 10^−2^(*M*_s_ − *T*_IT_)]. (6)

The initial-retained austenite fractions of TBF, TBM, and TM steels are *f*γ_0_ = 11.4, 7.2, and 5.5 vol.%, and the initial carbon concentrations are *C*γ_0_ = 0.65, 1.08 and 0.45 mass%, respectively (Table 2). The products of *f*γ_0_ and *C*γ_0_ of TBF, TBM, and TM steels are 0.074, 0.078, and 0.024, respectively. The product of TM steel is considerably low. Most of the retained austenite is located along the lath boundaries of bainitic ferrite and primary martensite and in the MA phase, in the same way as Ref. [10]. The volume fractions of the MA phase are *f*_MA_ = 2.0, 10.8, and 15.8 vol.% in TBF, TBM, and TM steels, respectively. Most of the MA phase mainly exists along these steels’ prior austenitic grain boundary and the lath boundaries of bainitic ferrite and primary martensite. The largest size of the MA phase is produced in TM steel. It seems that the prior austenitic grain size is the same for all three steels because of the same austenitizing temperature.

Vickers hardnesses of TBF, TBM, and TM steels are HV0.1 = 350, 405, and 422, respectively (Table 2). The HV0.1 of TBM steel is slightly lower than that of TM steel.

### 3.2. Strain-Hardening Behavior

#### 3.2.1. Flow Stress, Mechanical Properties, and Strain-Hardening

**Figure 5:**
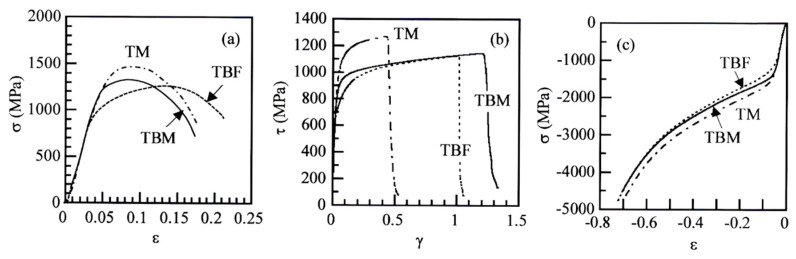
(**a**) Engineering tensile stress−strain (*σ−ε*) curves, (**b**) shear stress−strain (*τ−γ*) curves, and (**c**) compressive stress−strain curves (*σ−ε*) in TBF, TBM, and TM steels.

Figure 5 shows the flow curves of TBF, TBM, and TM steels deformed in tension, torsion, and compression. The mechanical properties are shown in Table 3. In tensile deformation, the tensile yield stress (YS) and tensile strength (TS) of TM steel are the highest. TBF steel possesses the largest uniform (UEl) and total (TEl) elongations. This is associated with high strain hardening in a large strain range. TBM and TM steels also have relatively large total elongations, although uniform elongations are considerably lower than that of TBF steel. It is noteworthy that the reductions of area (RAs) of TBM and TM steels are higher than that of TBF steel.

In torsional deformation, TM steel has the highest torsional shear yield stress (*τ*_0_) and maximum shear stress (*τ*_max_), with the lowest total shear strain. Differing from tensile deformation, TBM steel has a higher total shear strain than TBF steel, with just higher shear stress than TBF steel.

In compressive deformation, TM steel has higher compressive yield stress (*σ*_0_) and flow stress than those of TBF and TBM steels, in the same way as the tensile and torsional deformations. The compressive flow stress of TBM steel is slightly higher than that of TBF steel.

**Figure 6:**
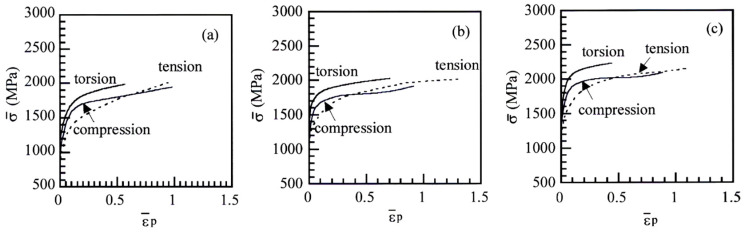
Equivalent stress–equivalent plastic strain ($\bar{\sigma }$−$\bar{\varepsilon }$_p_) curves of (**a**) TBF, (**b**) TBM, and (**c**) TM steels plastically deformed in tension, torsion, and compression.

Figure 6 shows the equivalent stress–equivalent plastic strain ($\bar{\sigma }-\bar{\varepsilon }$_p_) curves of TBF, TBM, and TM steels. Notably, the equivalent strain-hardening rate in an early strain range is higher in tension, compared to in torsion and compression. The $\bar{\sigma }-\bar{\varepsilon }$_p_ curves in torsion tend to be higher than those in tension and compression in all steels, in the same way as previously presented TRIP-aided polygonal ferrite (TPF) and TAM steels [21]. This is because the von Mises criterion was applied to calculate the $\bar{\sigma }-\bar{\varepsilon }$_p_ curves.

#### 3.2.2. X-ray Half-Width and Equivalent Plastic Strain Relation

**Figure 7:**
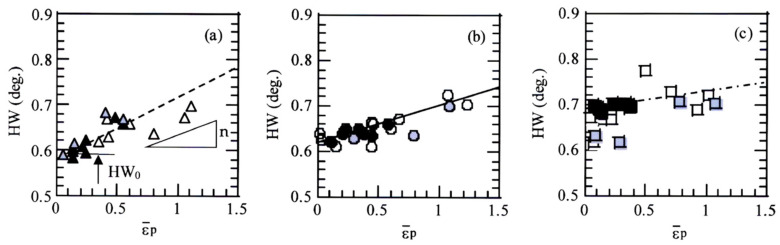
Relationship between X-ray half-width (HW) and equivalent plastic strain ($\bar{\varepsilon }$_p_) in (**a**) TBF (triangle marks), (**b**) TBM (circle marks), and (**c**) TM (square marks) steels plastically deformed in tension (open marks), torsion (black solid marks), and compression (gray solid marks).

**Figure 8:**
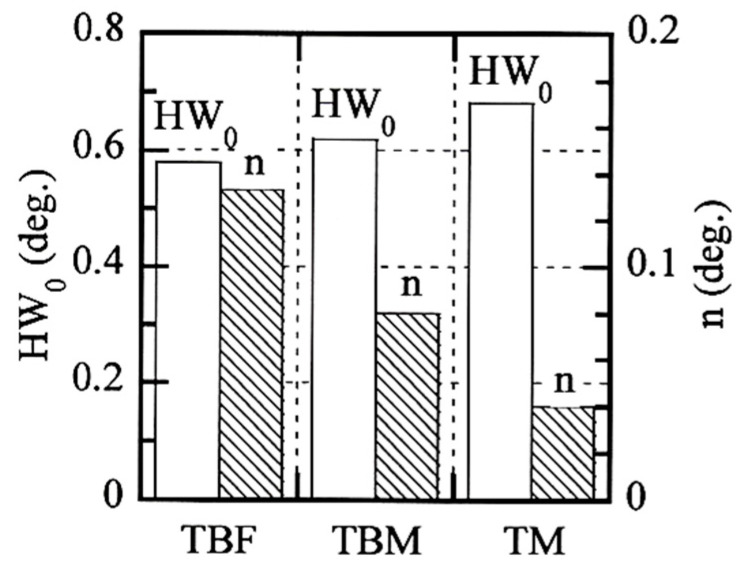
Comparison of HW_0_ and *n*-value in TBF, TBM, and TM steels. HW_0_ and *n*-value of TBF steel are decided in an equivalent plastic strain range of $\bar{\varepsilon }$_p_ = 0 to 0.6.

Figure 7 shows the X-ray half-width and equivalent plastic strain (HW−$\bar{\varepsilon }$_p_) relations in TBF, TBM, and TM steels plastically deformed in tension, torsion, and compression. The half-width linearly increases with increasing equivalent plastic strain in all steels. When the half-width characteristics are quantified by the half-width at $\bar{\varepsilon }$_p_ = 0 (HW_0_) and the slope of the straight line (*n*-value), TM steel has the highest HW_0_ (0.68 deg) and the smallest *n*-value (0.04), as shown in Figure 8. On the other hand, TBF steel exhibits the lowest HW_0_ (0.58 deg.) and the largest *n*-value (0.133). In this case, the *n*-value of TBF steel was measured in an equivalent strain range below $\bar{\varepsilon }$_p_ = 0.6. The HW_0_ and *n*-values of TBM steel are between those of TBF and TM steels. These HW_0_s and *n*-values are higher and lower than those of TPF and TAM steels, respectively [21]. Linear relationships in Figure 7 agree with a modified Williamson–Hall equation [22,23]. The HW_0_ and *n*-value may be correlated with the yield stress and strain-hardening rate in all steels, respectively. The relation between these HW_0_ and *n*-value and valuables in the modified Williamson–Hall equation will be investigated in the future.

### 3.3. Strain-Induced Martensite Transformation Behavior

**Figure 9:**
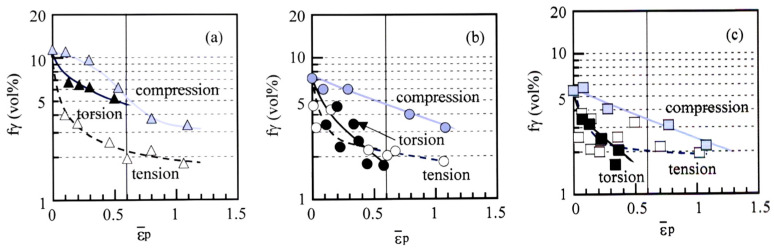
Variations in volume fraction of untransformed retained austenite (*f*γ) as a function of equivalent plastic strain ($\bar{\varepsilon }$_p_) in (**a**) TBF (triangle marks), (**b**) TBM (circle marks), and (**c**) TM (square marks) steels. Open marks: tension; black solid marks: torsion; gray solid marks: compression.

Figure 9 shows the variations in the volume fraction of untransformed retained austenite as a function of equivalent plastic strain in TBF, TBM, and TM steels. The strain-induced martensite transformation is most suppressed in compressive deformation in all steels. Tensile deformation promotes the strain-induced martensite transformation, especially in TBF steel. In TBM and TM steels, the strain-induced martensite transformation in tension is slightly promoted compared to in torsion (or zero mean normal stress).

**Figure 10:**
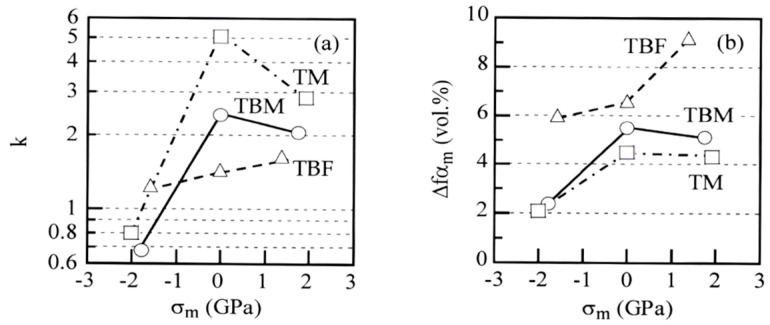
Variations in (**a**) *k*-value and (**b**) strain-induced martensite fraction (Δ*f*α_m_) as a function of mean normal stress (*σ*_m_) in TBF (triangle marks), TBM (circle marks), and TM (square marks) steels. The *k*-value was calculated in an equivalent plastic strain range of $\bar{\varepsilon }$_p_ = 0 to 0.6. The *k*-value and Δ*f*α_m_ of TM steel deformed in torsion are decided between $\bar{\varepsilon }$_p_ = 0 and 0.3.

Figure 10 shows the relationships between the *k*-value (the strain-induced transformation factor [10]) and mean normal stress and between strain-induced martensite fraction and mean normal stress in TBF, TBM, and TM steels. The *k*-value means the mechanical stability of the retained austenite and is defined by

(7) $$k\;=\;(\ln\;f\gamma_{0}\;-\;\ln\;f\gamma )/{\bar{\varepsilon }}_{P}$$

where *f*γ is the volume fraction of retained austenite in the steels subjected to an equivalent plastic strain of $\bar{\varepsilon }$_p_. When the *k*-values were measured in a range of $\bar{\varepsilon }$_P_ = 0 and 0.6 (Figure 9), the *k*-value approximately increases with increasing mean normal stress in all steels (Figure 10a). In this case, the *k*-value of TM steel deformed in torsion is decided between $\bar{\varepsilon }$_P_ = 0 and 0.3 (Table 2), because the TM steel fractured at the equivalent plastic strain below $\bar{\varepsilon }$_P_ = 0.6 (Figure 6c). The *k*-values in tension and torsion in TM steel are higher than those in TBF and TBM steels. On the other hand, when the strain-induced martensite fraction (Δ*f*α_m_) is defined by a difference between *f*γ_0_ and *f*γ at $\bar{\varepsilon }$_P_ = 0.6 (Figure 9), the strain-induced martensite fraction increases with increasing mean normal stress (Figure 10b). In addition, TBF steel has the highest strain-induced martensite fraction. On the other hand, TM steel exhibits the smallest strain-induced martensite fraction. The strain-induced martensite fraction of TBM steel is slightly higher than that of TM steel.

### 3.4. Micro-Void Initiation Behavior

**Figure 11:**
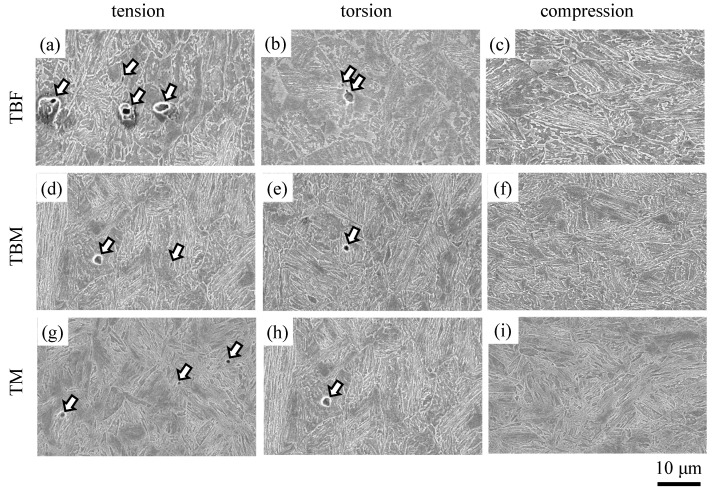
FE-SEM images of micro-voids initiated in (**a**–**c**) TBF, (**d**–**f**) TBM, and (**g**–**i**) TM steels plastically deformed to $\bar{\varepsilon }$_p_ = 0.6 in (**a**,**d**,**g**) tension, (**b**,**e**,**h**) torsion, and (**c**,**f**,**i**) compression. Arrows denote the micro-voids.

Figure 11 shows FE-SEM images of micro-voids in TBF, TBM, and TM steels plastically deformed to an equivalent plastic strain of $\bar{\varepsilon }$_p_ = 0.6. In this case, micro-voids larger than 0.5 μm are counted. It is found that no micro-voids are formed on compressive deformation. The most frequent micro-void initiation occurs by plastic deformation in tension. Many micro-voids initiate at the lath boundaries of bainitic ferrite and primary martensite, at the interfaces of the MA phase/matrix structure, and at the strain-induced martensite/matrix structure in all steels.

**Figure 12:**
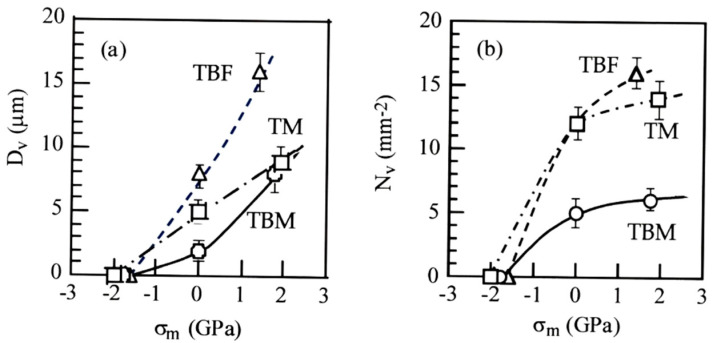
Variations in (**a**) the mean size (*D*_v_) and (**b**) mean number of per unit area (*N*_v_) of micro-voids are a function of mean normal stress (*σ*_m_) in TBF, TBM, and TM steels subjected to an equivalent plastic strain of $\bar{\varepsilon }$_p_ = 0.6.

Figure 12 shows the variations in the mean size (*D*_v_) and mean number per unit area (*N*_v_) of micro-voids as a function of mean normal stress in TBF, TBM, and TM steels plastically deformed to $\bar{\varepsilon }$_p_ = 0.6. The mean size and mean number of micro-voids increase with increasing mean normal stress. TBF steel has the maximum mean size and mean number of micro-voids. TBM steel shows the minimum mean size of micro-voids, and TM steel shows the minimum mean number of micro-voids, although the differences in the mean size and mean number between TBM and TM steels are small. Toji et al. [12] also showed a similar result using 0.19C-1.5Si-2.9Mn Q&P and TBF steels. Namely, in the Q&P steel with a mixed-matrix structure of bainitic ferrite and primary martensite, the micro-void initiation in tension was suppressed in comparison with that of TBF steel.

## 4. Discussion

In general, the flow stress of TRIP-aided steel can be decided by the sum of the following items (i) to (iv) [21]:

(i) “Flow stress of matrix structure”, including strain hardening.
(ii) “Long-range internal stress hardening”, which results from the difference in plastic strain between the matrix structure and second phase (retained austenite, strain-induced martensite, MA phase, etc.) [24].
(iii) “Strain-induced transformation hardening”, which results from an increase in strain-induced martensite fraction. The transformation also relaxes the localized stress concentration through an expansion strain [25]. In an early stage, the expansion strain brings on an initial yielding or continuous yielding.
(iv) “Forest dislocation hardening”, which is estimated by the Ashby equation [26].
  On the other hand, the micro-void initiation behavior is controlled by [10,14].
(v) “Matrix structure”: acicular or lath-type structure suppresses the void formation, compared to granular structure, by refining the structure size [10,12,14].
(vi) “Retained austenite characteristics”: a large amount of mechanically stable retained austenite suppresses the micro-void initiation due to the plastic relaxation by expansion strain on the strain-induced martensite transformation [10,12,14].
(vii) “A strength ratio” or a ratio of the second phase strength to the matrix structure strength: a high strength ratio increases the localized stress concentration and promotes void initiation at the matrix/second phase interface. Carbon-enriched strain-induced martensite enhances the strength ratio [12,27,28].

In the following, the effects of mean normal stress and microstructural properties on the deformation properties such as the strain hardening, strain-induced martensite transformation, and micro-void initiation behaviors in TBF, TBM, and TM steels are discussed considering items (i) to (vii).

### 4.1. Effect of Mean Normal Stress on Deformation Properties

In Figure 10, the *k*-values and Δ*f*α_m_ increased with increasing mean normal stress in all steels, although these levels differed in each steel. According to Hiwatashi et al. [29], stretch forming (an equi-biaxial tension or positive mean normal stress) significantly enhanced the strain-induced martensite transformation of the retained austenite in 0.11C-1.18Si-1.55Mn TPF steel. On the other hand, shrink flanging (a compression or negative mean normal stress) suppressed the strain-induced martensite transformation, and uniaxial tension slightly suppressed the strain-induced martensite transformation, compared to the stretch forming. They explained these results as follows. A positive mean normal stress assists the strain-induced martensite transformation because of the expansion stress or strain. Kawata et al. [19] also reported that compression stress suppressed the strain-induced transformation compared to tensile stress in 0.1C-1.2Si-1.5Mn and 0.2C-1.2Si-2.0Mn TPF steels. Therefore, high *k*-values and Δ*f*α_m_ under positive mean normal stress (Figure 10) are considered to be caused by high expansion stress or strain as proposed by Hiwatashi et al. [29].

As shown in Figure 12, micro-void initiation was promoted by a positive mean normal stress in all steels, particularly in TBF steel. This is because the positive mean stress originates the expansion stress which facilitates the micro-void initiation at the matrix/second phase interface, in the same way as the above strain-induced martensite transformation.

In Figure 6, the equivalent strain-hardening rate in an early strain range of tensile deformation was much higher than those of torsional and compressive deformation in all steel. The *k*-value in tension was higher than those in torsion and in compression in all steels (Figure 10a). Considering items (i) to (iv), the high equivalent strain-hardening behavior in tension may be associated with easy strain-induced martensite transformation, which promotes initial or continuous yielding [25], although the strain-induced martensite fraction differed in each steel (Figure 10b).

### 4.2. Effects of Microstructural Properties on Deformation Properties

In Figure 5a, TBF steel had a high strain-hardening rate in a large strain range, compared to TBM and TM steels. Considering items (i) to (iv) and the results of Figure 10, the high strain-hardening rate may be mainly associated with the large strain-induced martensite transformation hardening due to a large amount of mechanically stable retained austenite, with a contribution of the high strain hardening of its bainitic ferrite matrix. It is considered that a large uniform elongation of TBF steel is associated with large strain hardening in a large strain range. On the other hand, TM steel exhibited a high flow stress and high strain-hardening rate in an early strain range (Figure 5a). This is considered to be mainly associated with the high initial strain-hardening rate due to (1) the high dislocation density of primary martensite matrix structure and (2) the high long-range internal stress hardening due to high MA phase fraction with a contribution of early strain-induced martensite transformation hardening. TBM steel had the intermediate flow stress and strain-hardening rate between TBF and TM steels. Notably, the refined matrix structure of TBM steel contributes to an increase in the flow stress, total elongation, and reduction of area.

In Figure 10, the *k*-values in tension and torsion of TM steel were higher than those of TBF and TBM steels. Generally, retained austenite stability is controlled by the carbon concentration, size, morphology, and matrix structure surrounding the retained austenite. So the high *k*-values of TM steel may be caused by the low carbon concentration of retained austenite (Table 2) and the high flow stress of the primary martensite matrix structure, although the size was relatively small, and the morphology was filmy. In this case, retained austenite stability in the MA phase is relatively high, because most of the retained austenite is surrounded by harder secondary martensite and is highly carbon-enriched compared to the retained austenite at the primary martensite lath boundary.

The mean size and mean number per unit area of micro-voids were the largest in TBF steel (Figure 12). On the other hand, TBM showed the minimum mean size of micro-voids, and TM steel showed the minimum mean number of micro-voids, although the differences between TBM and TM steels were small. Considering items (v) to (vii), the easy micro-void initiation behavior of TBF steel may be associated with a relatively coarse soft matrix structure and a high strength ratio resulting from a large quantity of strain-induced martensite, although the strain-induced martensite transformation plays a role in lowering the localized stress concentration at the matrix structure/second phase interface. The suppressed micro-void initiation behavior of TBM steel is considered to be related to the refined mixed-matrix structure of bainitic ferrite/primary martensite and relatively stable retained austenite, despite a large MA phase fraction. Meanwhile, the suppressed micro-void initiation behavior of TM steel may be caused by a high uniformity of the primary lath-martensite matrix structure and the low strength ratio, despite the low volume fraction and mechanical stability of retained austenite and a large amount of MA phase.

## 5. Conclusions

The effect of mean normal stress on the deformation properties such as the strain-hardening, strain-induced martensite transformation, and micro-void initiation behaviors of TBF, TBM, and TM steels was investigated to evaluate the various cold formabilities. In addition, the deformation properties were related to the microstructural properties such as the matrix structure, retained austenite characteristics, and second-phase properties. The main results are summarized as follows:

(1) The positive mean normal stress increased the strain-hardening rate in an early strain range in all steels. This was mainly caused by facilitated strain-induced martensite transformation in an early strain range, resulting in an initial yielding or a continuous yielding.
(2) The equivalent plastic strain was linearly related to the X-ray half-width in all mean normal stress, which enabled the estimation of the equivalent stress in press-formed products. In this case, TBF steel had the lowest Vickers hardness and the highest *n*-value. On the other hand, TM steel exhibited the highest Vickers hardness and the lowest *n*-value.
(3) The positive mean normal stresses promoted the strain-induced martensitic transformation because of expansion strain. The strain-induced martensite transformation behavior of TM steel was promoted compared to TBF and TBM steels, although the transformation fraction was the smallest.
(4) The positive mean normal stress promoted the micro-void initiation by developing the expansion stress/strain, especially in TBF steel. The effect of the mean normal stress on the micro-void initiation behavior was small in TBM and TM steels. This was associated with (1) the mixed-matrix structure of bainitic ferrite and primary martensite structure and a relatively stable retained austenite and (2) the high uniformity of primary martensite matrix structure and a low strength ratio for TBM and TM steels, respectively.

## Figures and Tables

**Table 1:** Chemical composition (mass%) and measured martensite-start (*M*_S_, °C) and -finish temperatures (*M*_f_, °C) of a steel slab.

C	Si	Mn	P	S	Al	Nb	Cr	Mo	N	Fe	*M*_s_	*M*_f_
0.18	1.48	1.49	0.004	0.003	0.043	0.05	1.02	0.20	0.001	bal.	407	292

**Table 2:** Retained austenite characteristics and martensite/austenite constituent (MA phase) properties of TBF, TBM, and TM steels.

Steel	*f*γ_0_ (vol.%)	*C*γ_0_	*k*			Δ*f*α_m_			*f*_MA_ (vol.%)	HV0.1
		(mass%)	Tension	Torsion	Comp.	Tension	Torsion	Comp.		
TBF	11.4 ± 1.2	0.65 ± 0.14	1.64	1.44	1.24	9.2	6.6	6.0	2.0 ± 0.3	350
TBM	7.2 ± 1.4	1.08 ± 0.22	2.05	2.41	0.68	5.1	5.5	2.4	10.8 ± 1.2	405
TM	5.5 ± 1.5	0.45 ± 0.20	2.84	5.08	0.80	4.3	4.5	2.1	15.8 ± 1.8	422

*f*γ_0_: retained austenite fraction, *C*γ_0_: carbon concentration of retained austenite, *k*: strain-induced transformation factor, Δ*f*α_m_: strain-induced martensite fraction, *f*_MA_: volume fraction of MA phase. The *k*-values were calculated in an equivalent plastic strain range of $\bar{\varepsilon }$_p_ = 0 to 0.6. The *k*-value and Δ*f*α_m_ of TM steel deformed in torsion were decided between $\bar{\varepsilon }$_p_ = 0 and 0.3, HV0.1: Vickers hardness.

**Table 3:** Mechanical properties of TBF, TBM, and TM steels.

Steel	YS	TS	UEl	TEl	RA	τ_0_	τ_max_	σ_0_
	(MPa)	(MPa)	(%)	(%)	(%)	(MPa)	(MPa)	(MPa)
TBF	709 ± 15	1276 ± 18	9.0 ± 0.8	17.7 ± 2.3	49.5 ± 2.8	932 ± 24	1981 ± 41	937 ± 14
TBM	1058 ± 35	1310 ± 38	3.8 ± 0.5	14.7 ± 3.4	69.9 ± 3.7	1206 ± 37	2021 ± 57	1125 ± 28
TM	1073 ± 46	1463 ± 52	4.5 ± 1.0	14.6 ± 3.8	63.5 ± 4.2	1251 ± 45	2174 ± 63	1227 ± 37

YS: tensile yield stress, TS: tensile strength, UEl: uniform elongation, TEl: total elongation, RA: reduction of area, *τ*_0_: torsional shear yield stress, *τ*_max_: torsional maximum shear stress, *σ*_0_: compressive yield stress.

## Data Availability

The original contributions presented in the study are included in the article; further inquiries can be directed to the corresponding author.
